# Supplementary material for: Coupling of H3K27me3 recognition with transcriptional repression through the BAH-PHD-CPL2 complex in Arabidopsis
Source: Nat Commun. 2020 Dec 4;11:6212. doi: 10.1038/s41467-020-20089-0 (PMC7718874; doi:10.1038/s41467-020-20089-0)
Supplement: Supplementary file 1 — Supplementary Figures and Tables [file 41467_2020_20089_MOESM1_ESM.pdf]

Supplementary Information

Coupling of H3K27me3 recognition with transcriptional repression through the BAH-PHD-CPL2 complex in *Arabidopsis*

Yi-Zhe Zhang[1,2†], Jianlong Yuan[1,2†], Lingrui Zhang[3†], Chunxiang Chen[1], Yuhua Wang[1], Guiping Zhang[1], Li Peng[1], Si-Si Xie[1,2], Jing Jiang[4], Jian-Kang Zhu[1,3]*, Jiamu Du[5]* and Cheng-Guo Duan[1,4]*

Supplementary Figure 1 to 13

Supplementary Table 1 to 2

Supplementary Figure 1

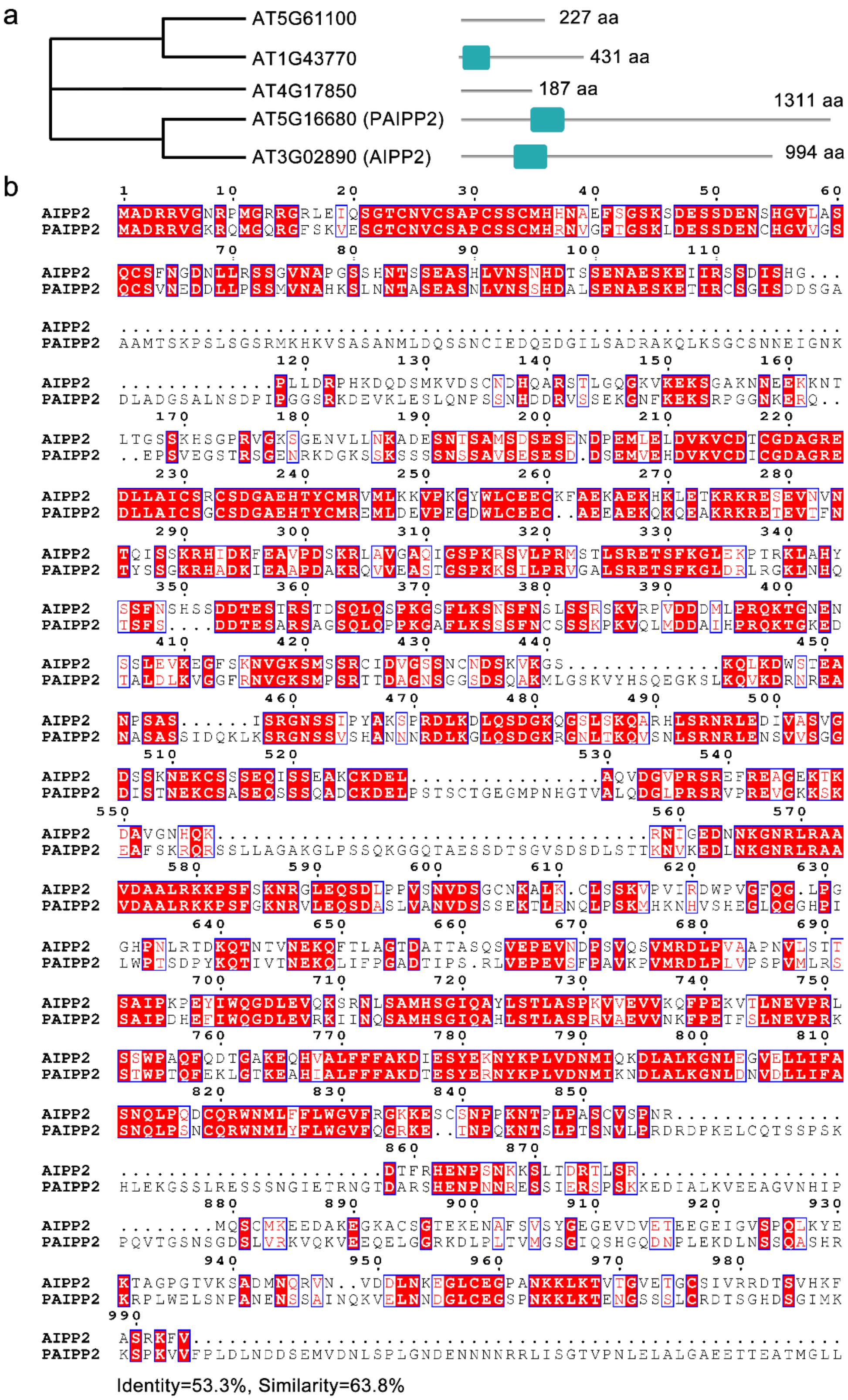

Supplementary Figure 1. PAIPP2 is the closest paralog of AIPP2 in *Arabidopsis.* a The phylogenetic tree between AIPP2 and its paralog proteins in *Arabidopsis* (left panel) and their domain structures. Green boxes indicate PHD domains. **b** The sequence alignment between AIPP2 and PAIPP2 showing the high similarity.

Supplementary Figure 2

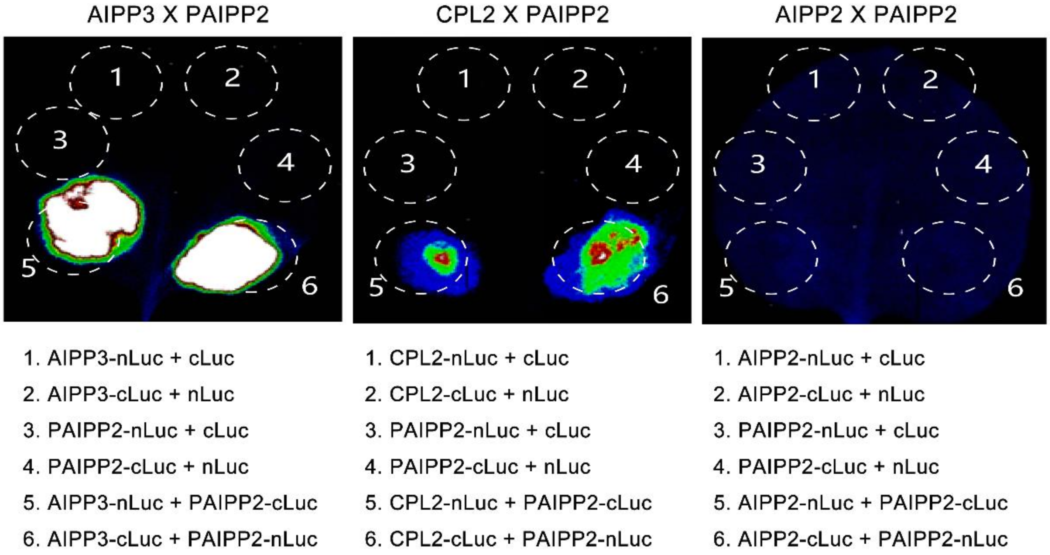

Supplementary Figure 2. Protein interactions revealed by split luciferase assay

Supplementary Figure 3

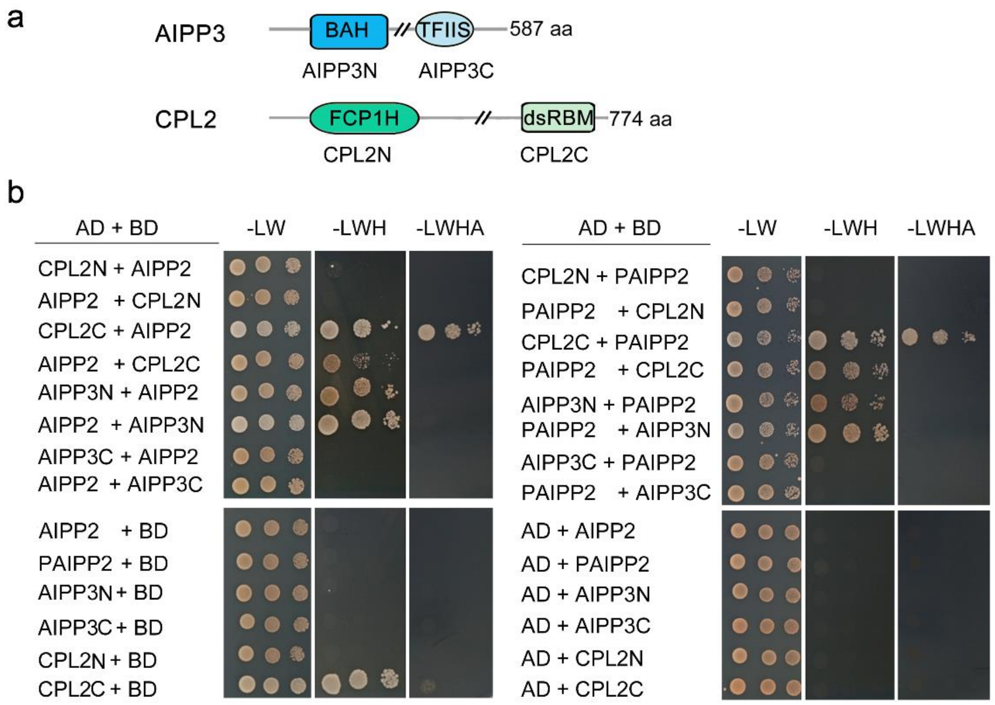

Supplementary Figure 3. Domain requirements for the interactions between BAH-PHD-CPL2 complex proteins. a The diagram showing the domains and truncated forms of different proteins. **b** Y2H assays showing the interactions between the truncated forms of proteins. N and C represent the N terminus and C terminus of the proteins, respectively.

Supplementary Figure 4

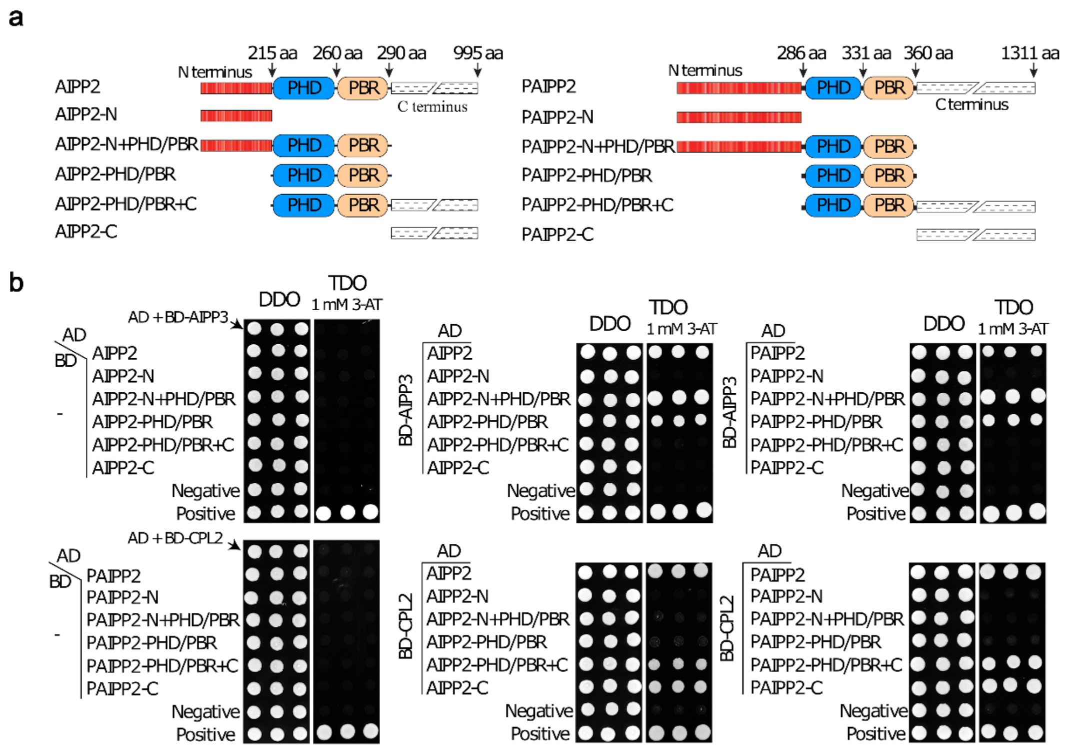

Supplementary Figure 4. Domain requirement for protein interactions. a Diagrams showing the split domain structure for protein interactions in b and c. **b** Yeast two-hybrid results showing the protein interactions of different combinations.

Supplementary Figure 5

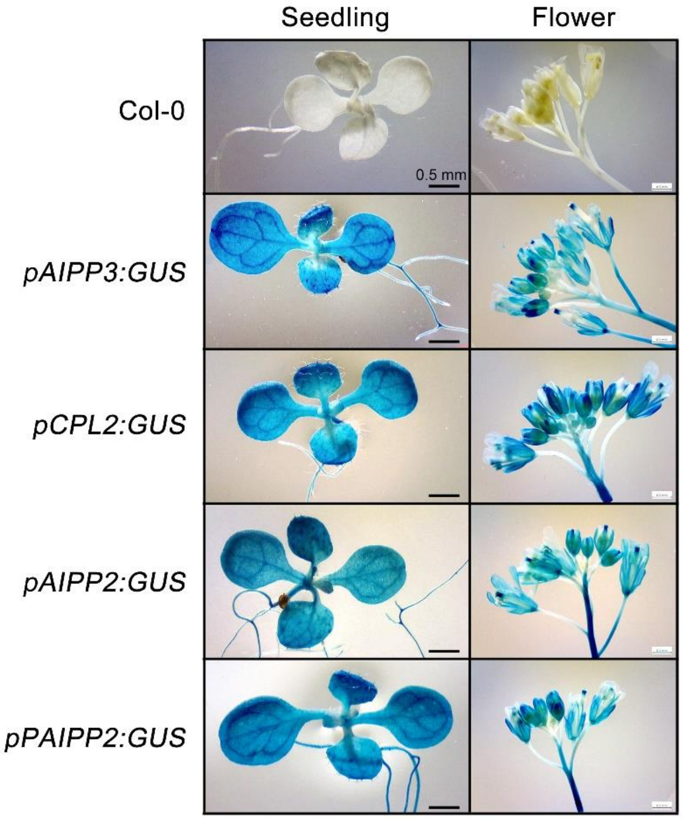

Supplementary Figure 5. Expression analysis of the *GUS* reporters from BAH-PHD-CPL2 complex genes in seedlings and inflorescence tissues. *GUS* reporter genes were expressed in transgenic *Arabidopsis* under the direction of native *AIPP3*, *AIPP2*, *PAIPP2* and *CPL2* promoters. The figure showing the GUS activity of seedlings and inflorescence tissues after being stained. Three independent experiments were performed and one representative result was shown.

Supplementary Figure 6

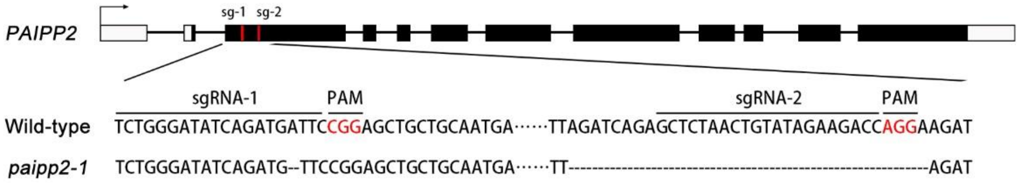

Supplementary Figure 6. CRISPR/Cas9-mediated mutagenesis of *PAIPP2.* The *paipp2-1* was generated by CRISPR/Cas9-mediated mutagenesis. Two sgRNAs were designed to target the N-terminal exon of *PAIPP2*. The *paipp2-1* mutation contains one nucleotide deletion and 33 bp deletion.

Supplementary Figure 7

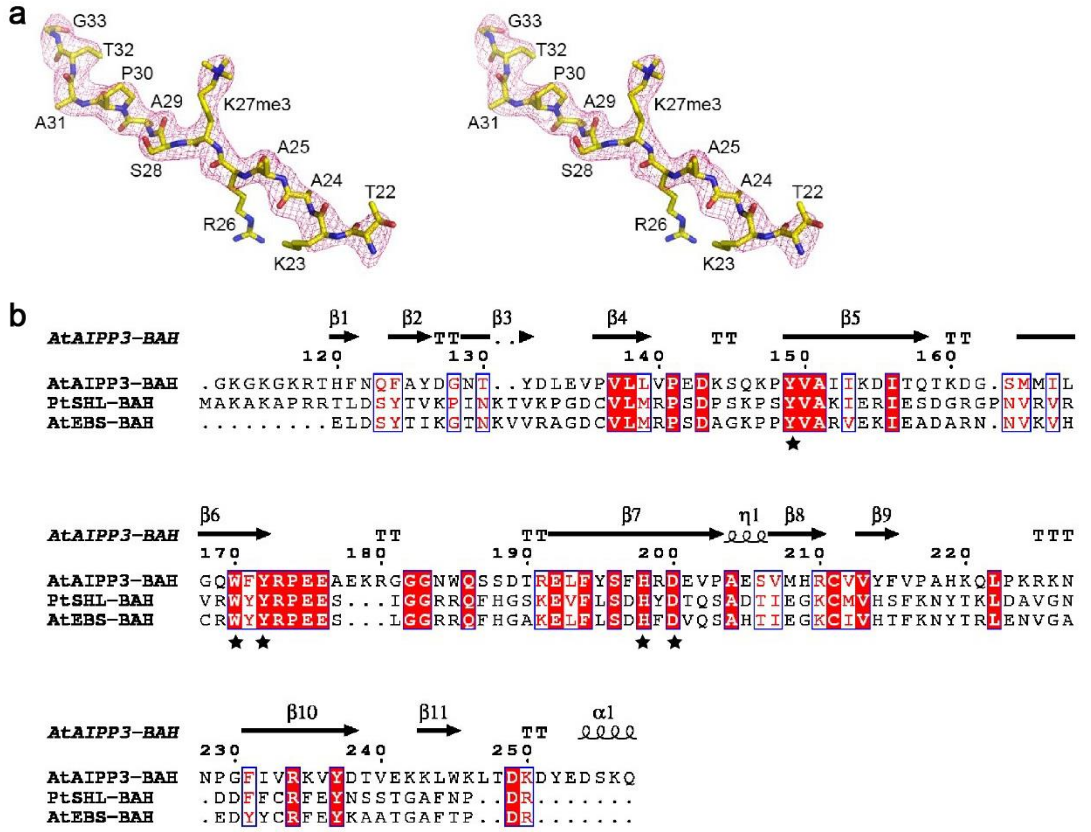

Supplementary Figure 7. Structural analysis of the AIPP3 BAH-H3K27me3 complex. a A stereo view of the SIGMAA-weighted 2Fo-Fc electron density map of the H3K27me3 peptide. **b** Structure-based sequence alignment of the BAH domains from *Arabidopsis* AIPP3 (AtAIPP3), *Populus trichocarpa* SHL (PtSHL), and *Arabidopsis* EBS (AtEBS) with the secondary structures of AtAIPP3 BAH showing on the top of the alignment. The conserved H3K27me3 peptide-interacting residues are highlighted by stars on the bottom.

Supplementary Figure 8

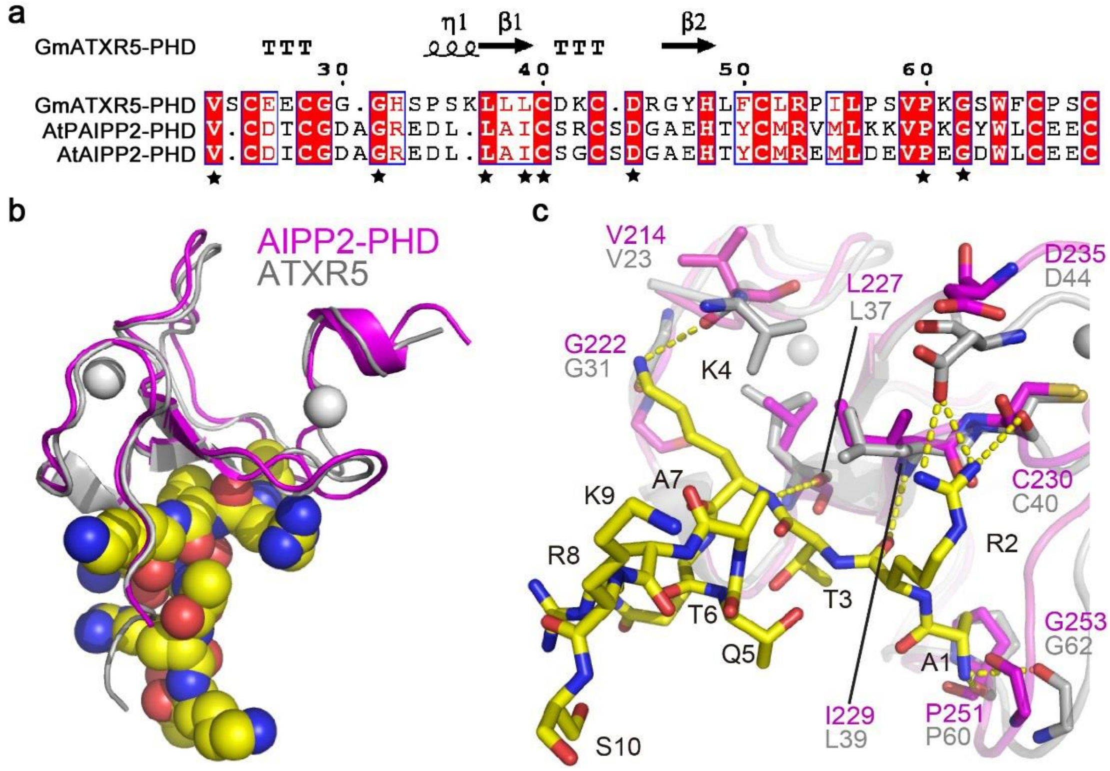

Supplementary Figure 8. Structural analysis of the AIPP2 PHD-H3 modeled complex. a Structure-based sequence alignment of the PHD fingers from *Arabidopsis* AIPP2 and PAIPP2 (AtAIPP2 and AtPAIPP2) and the modeling template *Glycine max* ATXR5 (GmATXR5) with the secondary structures of GmATXR5 showing at the top of the alignment. The conserved H3 peptide-interacting residues are highlighted with stars on the bottom. **b** The overall modeled structure of the AIPP2 PHD finger (in magenta) in complex with the H3 peptide (in the space-filling model) is overlaid with the structure of the model template ATXR5 PHD finger (in silver, PDB code: 5VAB). **c** The detailed interaction between the AIPP2 PHD finger and the H3 peptide with the interacting residues highlighted in the stick model and the hydrogen bonds highlighted in dashed yellow lines. The corresponding residues of the model template ATXR5 PHD finger are overlain, showing that almost all the peptide-binding residues are conserved.

Supplementary Figure 9

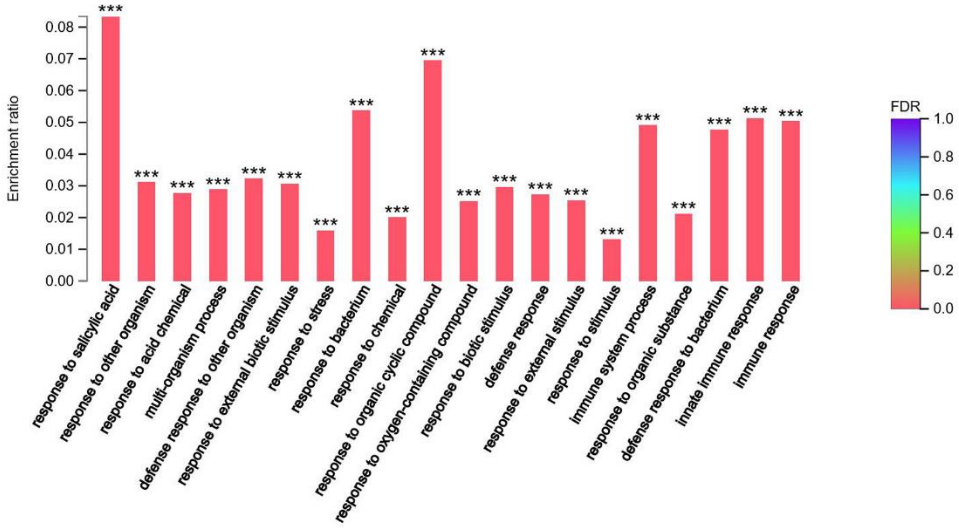

Supplementary Figure 9. FDR analysis of commonly up-regulated genes in the mutants of the BAH-PHD-CPL2 complex. Commonly up-regulated genes were assigned into different GO terms (x-axis). The y-axis indicates the ratio of the up-regulated gene number and the number of genes annotated in this pathway. The color indicates significance of enrichment. Fisher's exact test corrected by Bonferroni method was performed and *** represents p value < 0.001.

Supplementary Figure 10

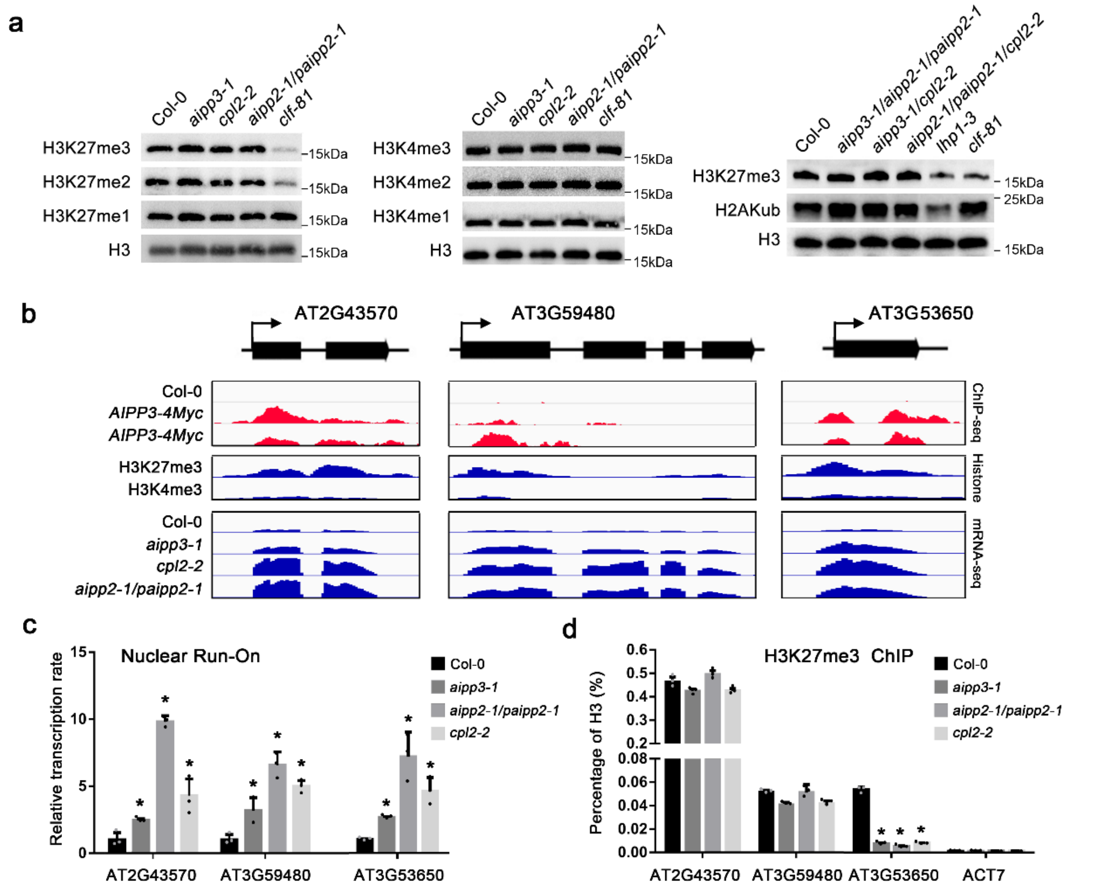

Supplementary Figure 10. The effects of BPC dysfunctions on the deposition of different histone marks. a The immunoblotting results showing the accumulation of the H3K27me1/2/3, H3K4me1/2/3 and H2Kub marks in the selected mutants. The H3 levels were determined to use as the loading controls. **b** Snapshots of IGV of AIPP3 ChIP-seq (upper panel), histone ChIP (middle panel) and mRNA-seq (lower panel) showing the distribution patterns of AIPP3, H3K27me3 and H3K4me3 at selected target genes, and the relative expression of these genes in different genotypes. **c** Nuclear Run-On showing the relative Pol II transcription rate at the selected target genes in Col-0 and *bpc* mutants. The relative transcription rate was normalized to *ACT2*. The data are the means ±S.D. of three biological repeats. Unpaired one-tailed *t*-test was performed and * represents p-value<0.01. **d** ChIP-qPCR results showing the relative occupancy of H3K27me3 at selected target genes in Col-0 and *bpc* mutants. The occupancy was first normalized to histone H3. The Data are the means ±S.D. of three biological repeats. Unpaired one-tailed *t*-test was performed and* represents p-value<0.01.

Supplementary Figure 11

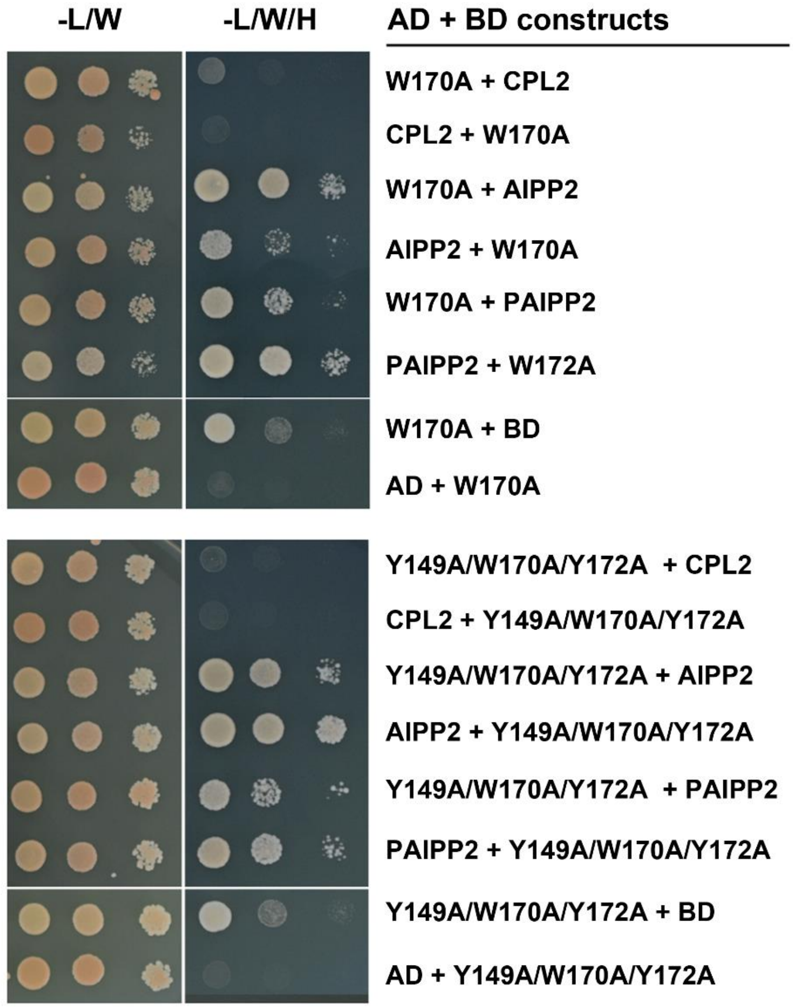

Supplementary Figure 11. The 170A and Y149A/W170A/Y172A mutations of AIPP3 did not affect its interaction with AIPP2 and PAIPP2. Y2H results showing the reciprocal interactions within the tested proteins. Yeast cultures with different protein combinations on SD-LW and SD-LWH are shown.

Supplementary Figure 12

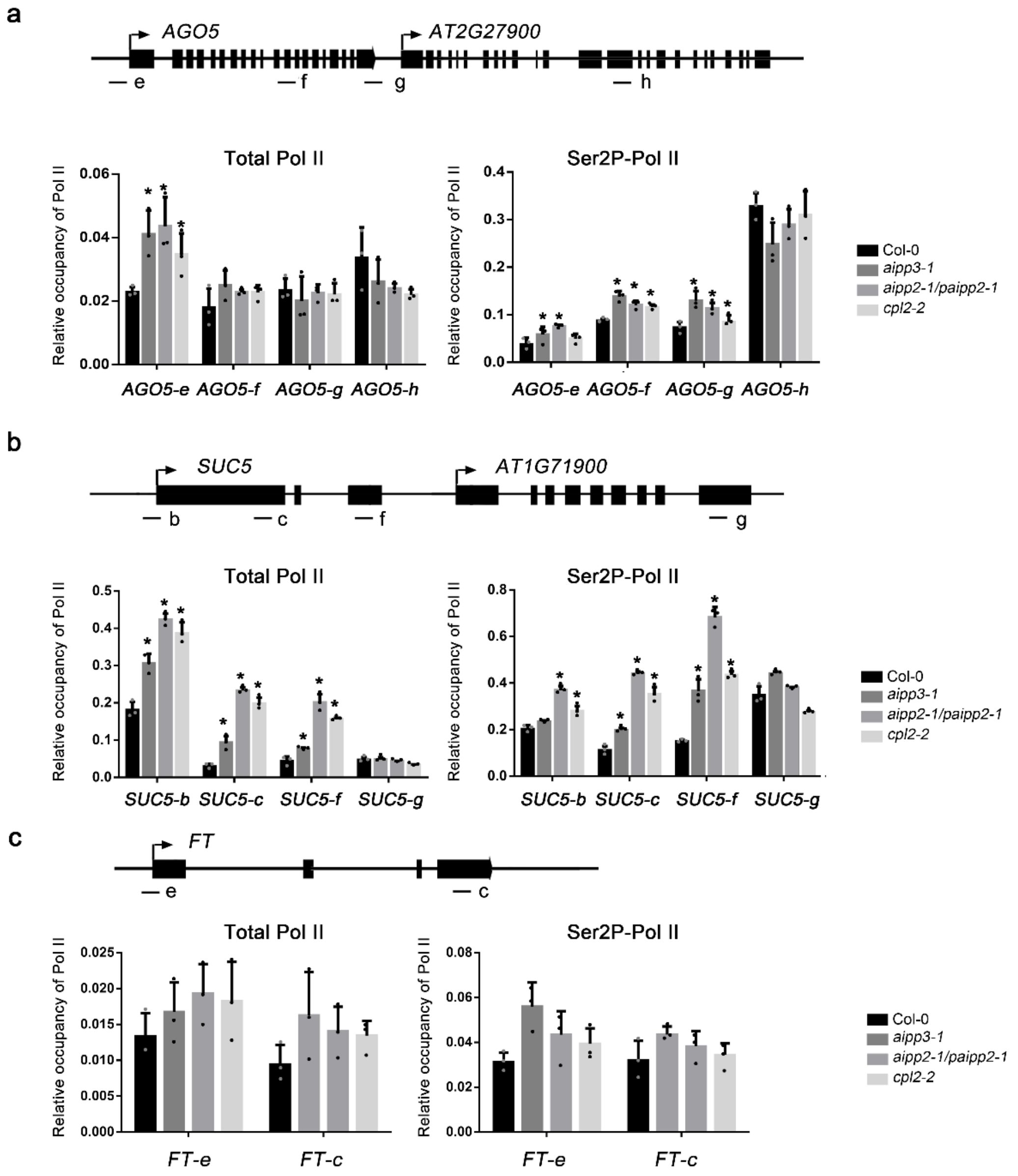

Supplementary Figure 12. Relative occupancy of unphosphorylated and Ser2P-Pol II at selected target genes. ChIP-qPCR showing the relative occupancy of unphosphorylated (total) and Ser2P-Pol II at *AGO5* (a), *SUC5* (b) and *FT* (c) loci. The occupancy was normalized to *ACT7*. The lowercase letters represent positions of ChIP-qPCR primers. The Data are the means ±S.D. of three biological repeats. Unpaired one-tailed *t*-test was performed and * represents p-value <0.05.

Supplementary Figure 13

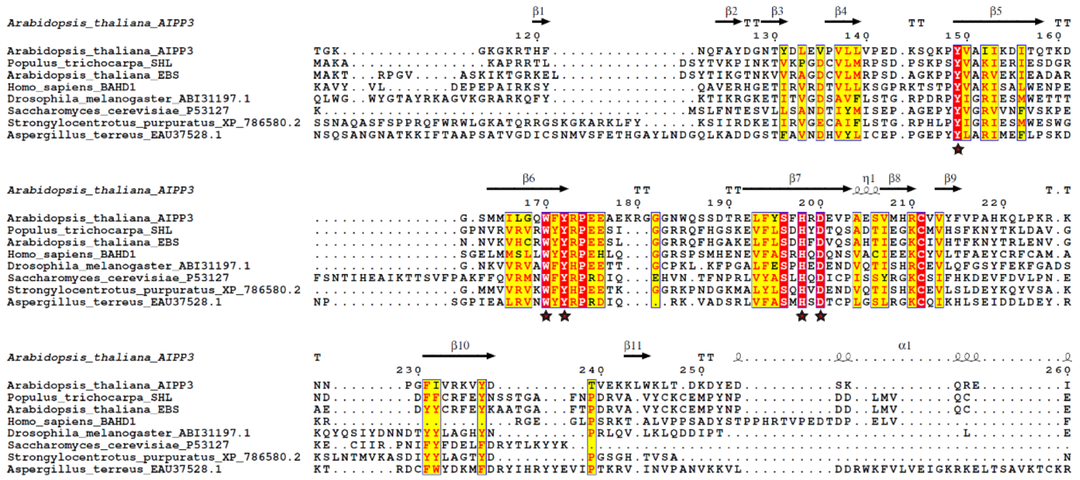

Supplementary Figure 13. A structure-based sequence alignment of potential H3K27me3 reading BAH domains from different species. The secondary structure of Arabidopsis AIPP3 BAH domain is on the top of the alignment. The conserved aromatic cage residues for methyl-lysine binding and histidine and aspartic acid residues for H3P30 binding are marked with stars.

Supplementary Table 1

Data collection and refinement statistics

	AIPP3-BAH-H3K27me3
**Data collection**	
Beamline	SSRF-BL19U1
Space group	$P3_121$
Wavelength (Å)	0.9789
Cell dimensions	
a=*b*, *c* (Å)	78.6, 72.7
Resolution (Å)	50.0-2.4 (2.44-2.40)[a]
R_{merge}	0.082 (1.121)
$I/\sigma I$	57.4 (2.8)
Completeness (%)	100.0 (100.0)
Redundancy	19.1 (18.7)
**Refinement**	
R_{work} / R_{free}	0.219 / 0.266
No. reflections	10,440
No. atoms	1,408
Protein / Peptide	1,314 / 83
Water / Tris	3 / 8
B-factors (Å^2)	90.7
Protein / Peptide	90.7 / 92.0
Water / Tris	63.3 / 93.6
R.m.s. deviations	
Bond lengths (Å)	0.008
Bond angles (°)	0.955

[a] Highest-resolution shell is shown in parentheses.

Supplementary Table 2. Primers used in this study

Primer	Sequence (5'-3')	Purpose
FLC-qF	GCAACGGTCTCATCGAGAAAGCT	RT-qPCR
FLC-qR	GATCATCAGCATGCTGTTTCCCAT	
FT-qF	GACCTCAGGAACTTCTATACTTTGGTTATG	
FT-qR	CTGTTTGCCTGCCAAGCTG	
SOC1-qF	GAGAAAGCTCTAGCTGCAGAA	
SOC1-qR	CTTGGGCTACTCTCTTCATCAC	
AGO5-qF	GAGTAAGCGAAGGGCAGTTTAG	
AGO5-qR	CACGAAAGTAACACGAGGAACA	
SUC5-qF	ATTGGATGGGTCGTGAAGTG	
SUC5-qR	CCTGAACTCCTTGGTCGTAAAG	
ACT2-qF	AGGTCCAGGAATCGTTCACA	RT /NRO-qPCR
ACT2-qR	GAGTTTGTCACACACAAGTGC	
18SrRNA-NRO-qF	TCCTAGTAAGCGCGAGTCATCA	NRO-qPCR
18SrRNA-NRO-qR	CGAACACTTCACCGGATCAT	
AGO5-NRO-qF	GAGTAAGCGAAGGGCAGTTTAG	
AGO5-NRO-qR	CACGAAAGTAACACGAGGAACA	
AT2G27900-NRO-qF	GAGAAGGAGACATGGACGAAAT	
AT2G27900-NRO-qR	GAGTGAGGGAATCTGGAAGAAC	
SUC5-NRO-qF	ATTGGATGGGTCGTGAAGTG	
SUC5-NRO-qR	CCTGAACTCCTTGGTCGTAAAG	
AT1G71900-NRO-qF	GTTGCGGATAAGCAAGCATATAA	
AT1G71900-NRO-qR	GTTATCGCAGTGGACCAGAA	
FT-NRO-qF	GGCCAAAGAGAGGTGACTAATG	
FT-NRO-qR	GGTCTTCTCCACCAATCTCAAC	
AT2G43570-F	GTGTCACCGTTCCTTCTAGTG	NRO/ChIP-qPCR
AT2G43570-R	GTTGCAGCAAATATGGCTACTG	
AT3G53650-F	AGCATCAATGGCTCCGAAA	
AT3G53650-R	CTTCTTCTCCGCCTTTGGTAA	
AT3G59480-F	CTGCATCTAACGGCGAGAAA	
AT3G59480-R	TTGATGAAGCCAGGAGCATC	
AGO5a-qF	CCTCCAATCGAATCCACAAGAT	ChIP-qPCR
AGO5a-qR	GTCCATAACATAACGTGCCAAATAG	
AGO5b-qF	GAAACGTCGGAAGAGGTGAA	
AGO5b-qR	AGACGAAGACGCAACAGAAA	
AGO5c-qF	GAGTAAGCGAAGGGCAGTTTAG	
AGO5c-qR	CACGAAAGTAACACGAGGAACA	
AGO5d-qF	GGCACTGGTGTCATGGATTTA	
AGO5d-qR	CTCAGGACGATCGGAGATAGAA	
AGO5e-qF	CCGGGAATGCAAAGAGATGA	
AGO5e-qR	AAACTGGTTACTTGTGTGTATTGTG	
AGO5f-qF	CCTGCTATTCCGTTCATCTCTT	

AGO5f-qR	GATCCAGTCACATCAGGCAATA	
AGO5g-qF	TTGAAGGATCTGGCCAACTT	
AGO5g-qR	TGAGCCAAATCATCGTCCAA	
AGO5h-qF	GAGAAGGAGACATGGACGAAAT	
AGO5h-qR	GAGTGAGGGAATCTGGAAGAAC	
SUC5a-qF	GACCGTCTATTTCACCCTTCTT	
SUC5a-qR	GTGTGATCTTCTGGGTCTTTCT	
SUC5b-qF	CTCCAATGTCTCATCTCCTACATC	
SUC5b-qR	CTCTAACGCCGTAGCATTGT	
SUC5c-qF	ATTGGATGGGTCGTGAAGTG	
SUC5c-qR	CCTGAACTCCTTGGTCGTAAAG	
SUC5d-qF	CCTAGTCTCACAGCAGGAATTA	
SUC5d-qR	GACCCAGACGAACCCTAAAT	
SUC5e-qF	GGCAACTTTGGCAAGTATGTT	
SUC5e-qR	TGTTACGCCAGCCTTGTT	
SUC5f-qF	CCAATGGGCCTTTCACTCTT	
SUC5f-qR	AGAACTGGCCTCATATTCCTTAATC	
SUC5g-qF	GTTGCGGATAAGCAAGCATATAA	
SUC5g-qR	GTTATCGCAGTGGACCAGAA	
FTa-qF	GTTCGGACATTGGTAGGTATGG	
FTa-qR	AAGGGATCCTTCAGGTTAGATAGA	
FTb-qF	GGCCAAAGAGAGGTGACTAATG	
FTb-qR	GGTCTTCTCCACCAATCTCAAC	
FTc-qF	GGAATTCATCGTGTCGTGTTT	
FTc-qR	GGAAGGCCGAGATTGTAGAT	
FTd-qF	TGGGATAAATACGAGGAACAACT	
FTd-qR	GATCTAACCATAACTTGACAGCATAAC	
FTe-qF	TTCACCGACCCGAGTTAATG	
FTe-qR	GGTGGTTTCTCTGTGTTGATTG	
ACT7-qF	CGTTTCGCTTTCCTTAGTGTTAGCT	
ACT7-qR	AGCGAACGGATCTAGAGACTCACCTTG	
AtSN1-qF	CCAGAAATTCATCTTCTTTGGAAAAG	
AtSN1-qR	GCCCAGTGGTAAATCTCTCAGATAGA	
