# Supplementary material for: Coupling of H3K27me3 recognition with transcriptional repression through the BAH-PHD-CPL2 complex in Arabidopsis
Source: Nat Commun. 2020 Dec 4;11:6212. doi: 10.1038/s41467-020-20089-0 (PMC7718874; doi:10.1038/s41467-020-20089-0)
Supplement: Supplementary file 3 — Descriptions of Additional Supplementary Files [file 41467_2020_20089_MOESM3_ESM.pdf]

Descriptions of Additional Supplementary Files

Supplementary Data 1

Description: IP-MS analysis

Supplementary Data 2

Description: Commonly up-regulated genes in BPC complex mutants
